# Supplementary material for: Tunable resistivity exponents in the metallic phase of epitaxial nickelates
Source: Nat Commun. 2020 Jun 11;11:2949. doi: 10.1038/s41467-020-16740-5 (PMC7289814; doi:10.1038/s41467-020-16740-5)
Supplement: Supplementary file 1 — Supplementary Information [file 41467_2020_16740_MOESM1_ESM.pdf]

Supplementary information for "Tunable resistivity exponents in the metallic phase of epitaxial nickelates"

Guo et al.

May 3, 2020

Supplementary Note 1: Characterization method

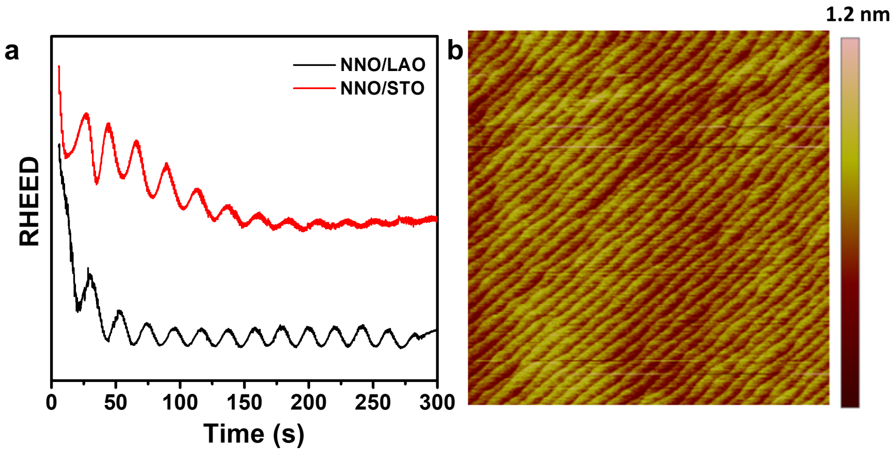

Supplementary Figure 1. Growth of nickelate films. (a) RHEED intensity oscillations observed *in-situ* during PLD deposition. (b) AFM topography image of a 5×5 μ m× μ m area in a 5 nm thick NNO film grown on a LAO substrate.

The structural and transport properties of all films were studied in detail. The in-situ RHEED patterns and atomic force microscopy (AFM) topography images were captured for the as-grown films, as shown in Supplementary Fig. 1 for one of the films. The intensity oscillations of NNO/LAO and NNO/STO films indicate that at least the first 13 layers ($\sim$ 5 nm) of NNO film are deposited atomic-layer by atomic-layer. Transport properties were measured between 5 K to 400 K by the van der Pauw method.[1]

To calculate the in-plane strain ($\varepsilon_{\rm xx}$), the in-plane lattice parameter (a) of each film is obtained by performing a reciprocal space map scanning around the (103)c peak. For this, the value of $k/2k_0$ at the center of the film peak is extracted and then the in-plane lattice parameter is calculated according to:

$$\frac{k}{2k_0} = \frac{\frac{2\pi}{a}}{2\frac{2\pi}{\lambda}} \tag{1}$$

Where λ is the wavelength of the X-ray (1.5406 Å in our case). The in-plane strain of the film is then calculated with respect to the unit cell volume of bulk $NdNiO_3$ as:

$$\varepsilon_{xx} = \frac{a - a_b}{a_b} \tag{2}$$

Where $a^{\rm b} = V^{1/3}$, being V the unit cell volume of bulk $NdNiO_3$. The $\varepsilon_{\rm xx}$ of all films is presented in Supplementary Table 1.

Table 1. The in plane strain ($\varepsilon_{\mathrm{xx}}$) of NNO films with different thickness grown on different substrates.

	5 nm	7.5 nm	10 nm	15 nm	20 nm	40 nm
NNO/LAO	-0.3 %	-0.29 %	-0.27 %	×	×	-0.20 %
NNO/NGO	+1.34 %	×	×	×	×	×
NNO/STO	+2.54 %	+2.48 %	+2.40 %	+2.11 %	+1.92 %	+1.52%
NNO/DSO	×	×	+3.86 %	×	×	×

Supplementary Note 2: Extracting the exponent n

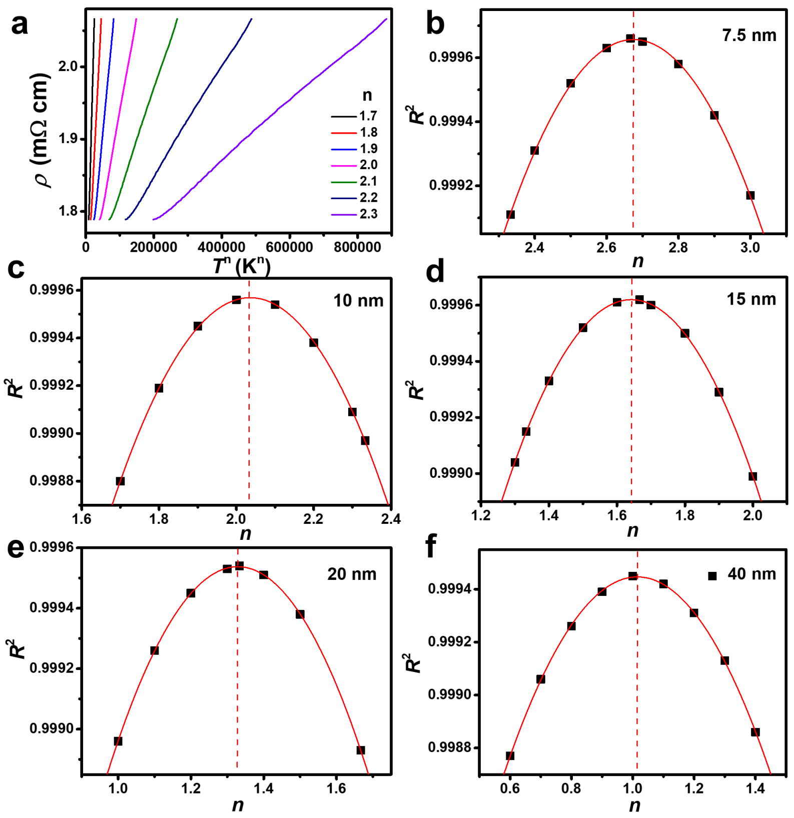

Supplementary Figure 2. Extraction of exponents (n). Resistivity of a 10 nm NNO/STO film as a function of T^{n} with different n values. (b-f) The coefficient of determination (R^2) as a function of n for NNO/STO films with thicknesses of 7.5 nm, 10 nm, 15 nm, 20 nm and 40 nm, respectively.

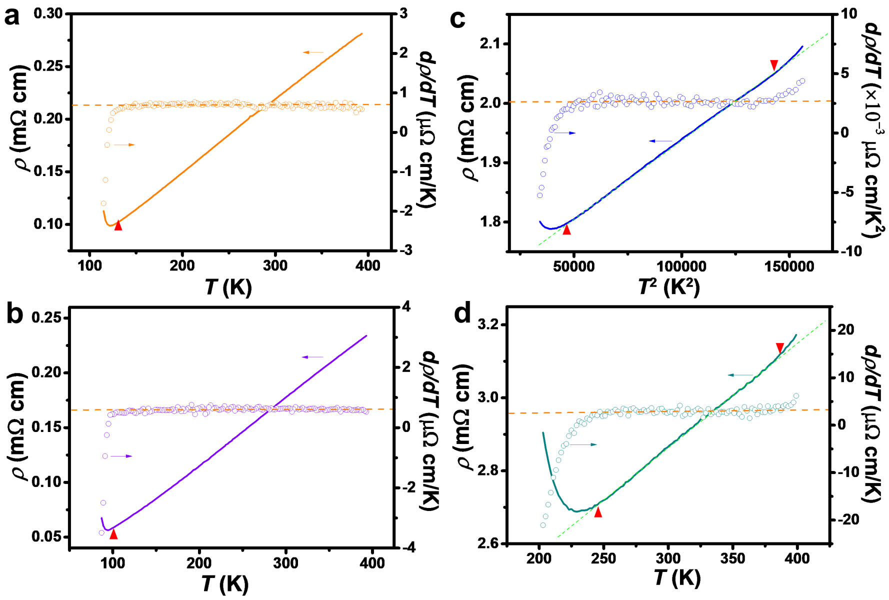

Supplementary Figure 3. Validity region. Resistivity (solid lines) and its temperature derivative (open circles) versus T^n for NNO/LAO films with thickness of 5 nm (a) and 7.5 nm (b) and for NNO/STO films with thicknesses of 10 nm (c) and 40 nm (d). The upturn in resistivity signalling the metal-insulator transition is visible. The red triangles signal the temperature region where the resistivity starts to deviate from the best fit.

It is important to pay attention to the extraction of the ρ(T) power law exponents from the experimental data. Proper determination of scaling exponents requires access to many scales in the experimental parameters. In the case of temperature scaling, this is not possible so the exponents obtained from the experimental resistivity-temperature analysis can be considered as *apparent* scaling exponents. In order to extract these exponents, the experimentally obtained ρ(T) of each film was plotted as a function of T^{n} for different n values, as shown for one of the films in Supplementary Fig. 2a. All the different curves were fitted as linear fits and the best fit (the best n) was determined by the largest R^2 factor. This was done by interpolating to the maximum of the R^2 parabola, as shown for several examples in Supplementary Fig. 2b-f.

The range of validity of the fits was checked by plotting the temperature derivative of ρ(T) as a function T^{n}. These plots can be seen for some of the films in Supplementary Fig. 3.

Supplementary Note 3: $NdNiO_3$ thin films on $NdGaO_3$ substrate

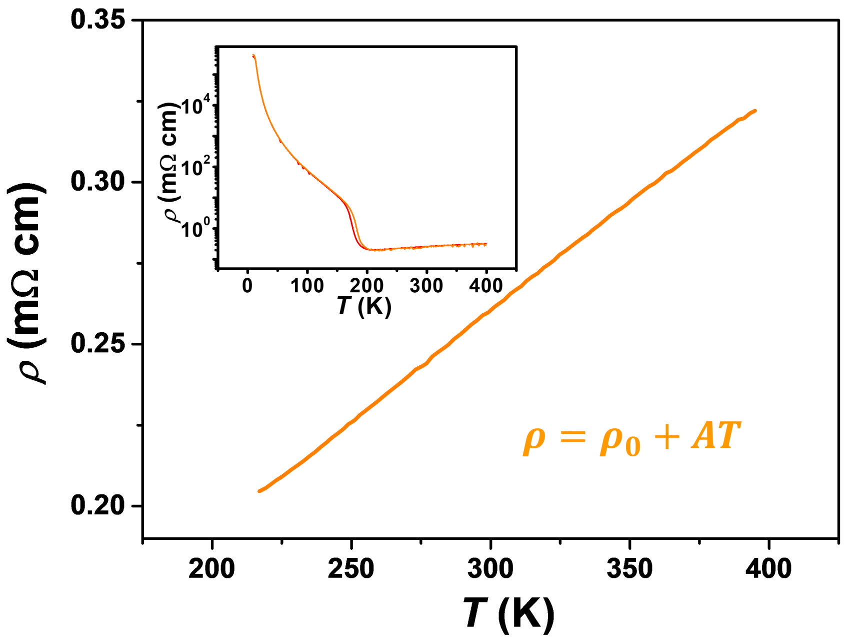

Supplementary Figure 4. Temperature-dependent resistivity of NNO films on NGO. T-linear dependent resistivity in the metallic phase of a 5 nm NNO/NGO film. Inset: Temperature dependence of resistivity in an extended temperature range, including both cooling and heating processes.

Next to the series of samples deposited on LAO and STO substrates, NNO films with 5 nm in thickness have been grown on $NdGaO_3$ substrates, imposing a nominal tensile strain value of + 1.34 %. These films show transport properties similar to those of the films on STO under similar tensile strain (see Supplementary Fig. 4).

Supplementary Note 4: X-ray Photoelectron Spectroscopy (XPS)

XPS characterization was conducted on a VG Microtech CLAM2 XPS system with a non monochromatic Al Kα source. All the measured spectra were fitted with a Shirley-type background, including a shift due to charging, using the Au $4f_{7/2}$ XPS peak position as reference. The Ni $2p_{3/2}$ peak position obtained from the fit is 854.86 eV for the 5 nm thick NNO/STO film. This energy is in between those expected for the Ni^{3+} peak (855.9 eV) and the Ni^{2+} peak

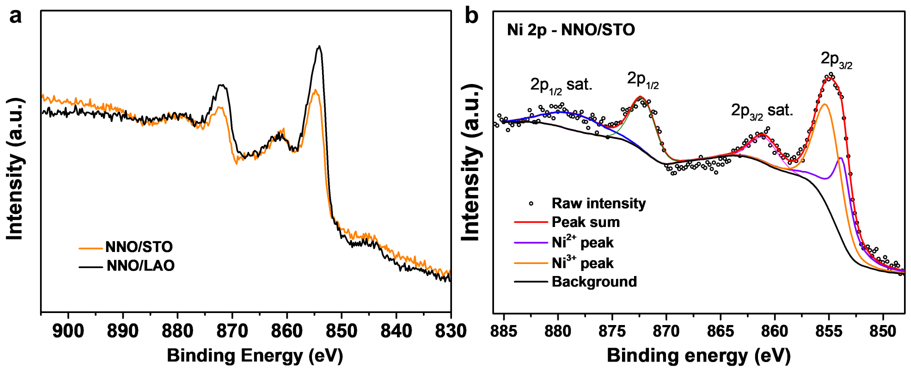

Supplementary Figure 5. XPS spectroscopy. (a) XPS spectra around the Ni 2p absorption edge of two 5 nm thick $NdNiO_3$ films grown on $SrTiO_3$ (orange line) and on $LaAlO_3$ (black line) substrates. (b) Spectra of the 5 nm thick $NdNiO_3$ film grown on $SrTiO_3$ showing the components of the fit.

(853.9 eV), indicating the coexistence of both oxidation states. However, due to the experimental accuracy of the measurements (± 0.8 eV), the extraction of an exact Ni^{3+}/Ni^{2+} ratio is impossible for us. In addition, the films on LAO also show coexistence of Ni^{2+} and Ni^{3+} peaks. This has also been reported by Preziosi et al.,[2] which showed that even the samples with the sharpest MIT and resistance changes of nearly 6 orders of magnitude still showed a Ni^{2+}/Ni^{3+} ratio of about 0.2. This makes the quantification of the relative Ni^{2+} in the different samples very challenging.

Supplementary Note 5: Effect of oxygen vacancies

To directly show the effect of oxygen vacancies on the resistivity, two 20 nm NNO films were grown on STO and LAO substrates, respectively, and their concentration of oxygen vacancies was tuned by changing the annealing conditions. At the first step, the two films were vacuum-annealed at a pressure of 10^{-7} mbar (V(a)) just after deposition, while cooling down from the deposition temperature with a rate of -5 °C/min. In this process, a large amount of oxygen vacancies is induced in the films. In the following process, the oxygen-deficient films were annealed gradually in an oxygen-enriched environment (O(a)) by increasing the temperature step-by-step. In this way, the oxygen vacancies are gradually refilled, resulting in a NNO film with tuned concentration of oxygen vacancies.

The corresponding changes in structure and electrical transport properties after the different annealing steps were characterized with X-ray diffraction and resistivity measurements, as shown in Supplementary Fig. 6. From Supplementary Fig. 6a and b, it is clear that the (002) diffraction peak of NNO films

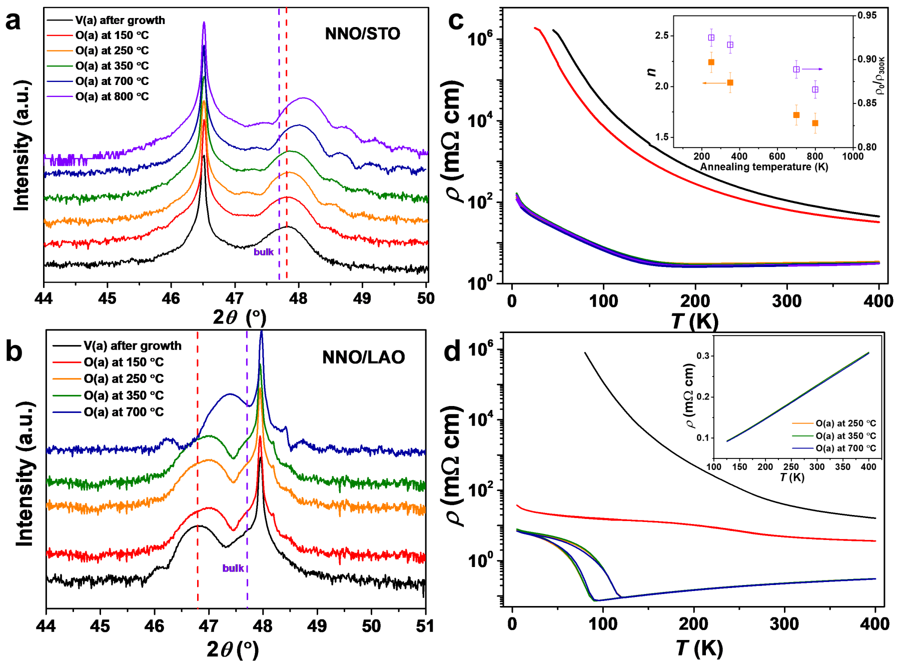

Supplementary Figure 6. Structural and electrical characterization of oxygen-deficient nickelate films. XRD $2\theta - \omega$ scans around the (002) peaks of a 20 nm NNO/STO (a) and NNO/LAO (b) film at various annealing stages. The red dashed lines indicate the (002) peak of NNO films after vacuum annealing (V(a)), while the purple dashed lines point out the peak position of bulk NNO. After annealing in an oxygen-enriched environment (O(a)), at increasingly larger temperatrues, the (002) peak shows a clear shift to larger angles. The resistivity as a function of temperature of the NNO/STO and NNO/LAO films for the various annealing temperatures are shown in (c) and (d), respectively. The inset of (c) shows the extracted exponents (left axis) and residual resistivity ratio (right axis) in the metallic phase of the NNO/STO film, as a function of annealing temperature. The error bar was determined from the fit to experimental data. The inset of (d) reveals the linear-T dependence of resistivity in the metallic phase of the NNO/LAO film after annealing.

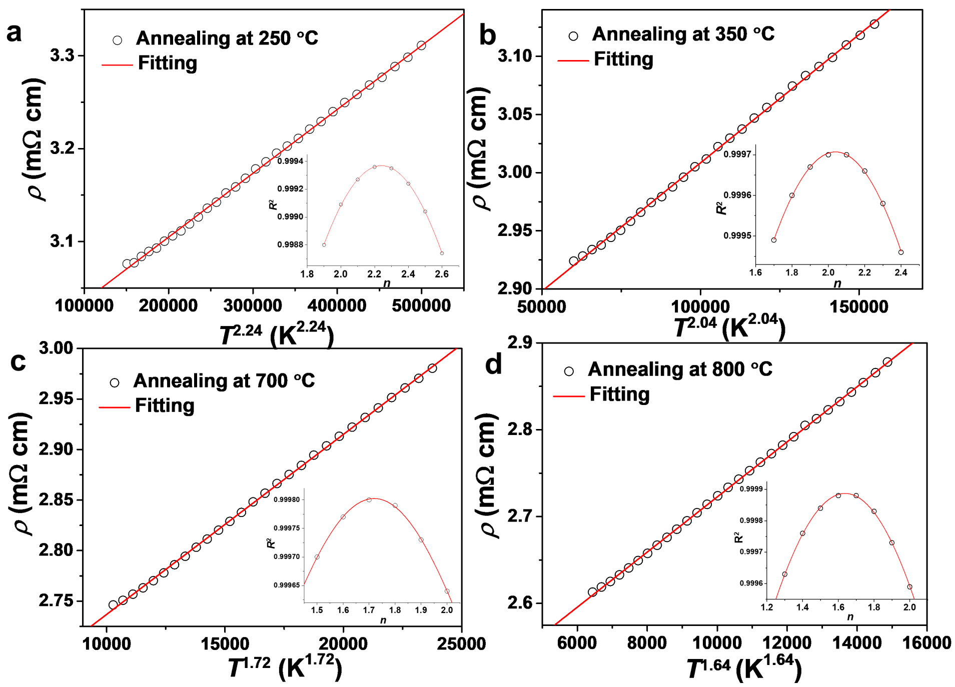

Supplementary Figure 7. Resistivity of oxygen-deficient nickelate films as a function of $T^{\mathbf{n}}$. Resistivity in the metallic phase of the 20 nm NNO/STO film as a function of temperature to the n-exponent extracted by maximizing R^2, as shown in the insets. The different panels show the curves after annealing at (a) 250 °C, (b) 350 °C, (c) 700 °C, (d) 800 °C. The inset shows the coefficient of determination (R^2) as a function of n in each state.

grown on both the STO and LAO substrates shows a shift to larger angle after annealing in oxygen gas and high temperature, corresponding to a decrease of out-of-plane lattice parameter (c). It is well known that the existence of oxygen vacancies gives rise to a enlarged unit cell volume of film. Therefore, the decrease of c manifests a gradual reduction of the density of oxygen vacancies. However, clear differeces are observed in both films: while a significant shift of the (002) peak could be observed in NNO/LAO film after annealing at 150 °C, a higher temperature is needed to observe changes in the NNO/STO film. This indicates the refilling of oxygen vacancies is more difficult in the tensile-strained NNO film than in the compressive-strained film, which agrees with our early discussions. Another clear difference between NNO/STO and NNO/LAO films is in the evolution of their resistivity with the annealing process. The resistivity of the NNO/LAO film shows a several order of magnitude reduction after annealing at 150 °C (see Supplementary Fig. 6d), however, a much less difference is observed in NNO/STO film after comparable treatment (see Supplementary Fig. 6c). A more significant difference in these two systems is found in their recovered metallic phase. As shown in the inset of see Supplementary Fig. 6d, the resistivity in the metallic phase of NNO/LAO film exhibits a robust linear temperature dependence, regardless of the change concentration of oxygen vacancies. On the contrary, the resistivity-temperature scaling in NNO/STO film shows a clear evolution with annealing process. The methods used for extracting exponents are the same with that discussed in Supplementary Note 2. The fitting of resistivity with extracted exponents at various states during the annealing of NNO/STO film is shown in Supplementary Fig. 7. The extracted exponents and residual resistivity ratio is summarized in the inset of Supplementary Fig. 6c. Clearly, both the exponents and residual resistivity ratio shows a decrease with the increasing annealing temperature, or in other words, with the reduce amount of oxygen vacancies. Hence, we reveal, in a direct manner, that the tunable exponents in the metallic phase of tensile-strained NNO films are strongly correlated with the degree of oxygen vacancies in their lattice.

Supplementary Note 6: $NdNiO_3$ thin films on $DyScO_3$ substrate

As mentioned in the main text, large enough tensile strain should induce a large density of defects that would, eventually, suppress the metallic phase. This is, indeed, shown in the inset of Supplementary Fig. 8a for film grown on DSO substrates (+3.86 % tensile strain). There is no metallic phase below 400 K. In this case, the resistivity is well described by a Mott's variable range hopping (VRH) conduction model[3] (see Supplementary Fig. 8b) for $T < 70$ K; while the data follows a simple thermal activation model with a single activation energy E_a= 32 meV (consistent with a near neighbours hopping conductions model, NNH) for temperatures above T= 70 K. This evolution from VRH to NNH with increasing temperature is characteristic of disordered solids.[4]

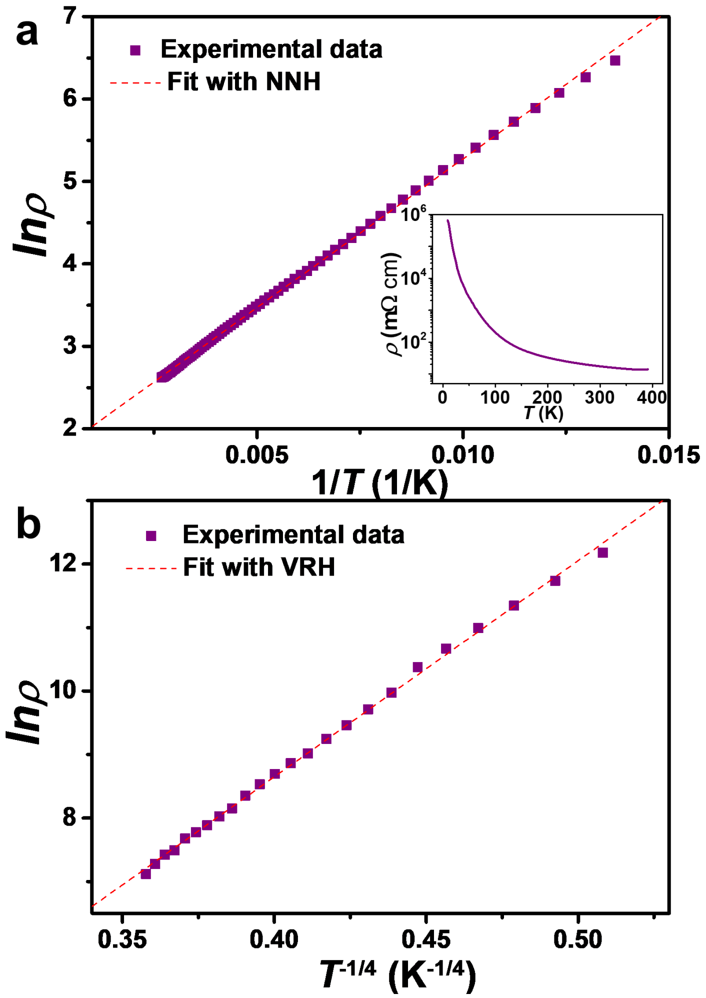

Supplementary Figure 8. Fit to resistivity of NNO/DSO. (a) $ln\rho$ versus T^{-1} for a 10 nm NNO/DSO film, together with the fit to a Near Neighbours Hopping (NNH) model (thermally-activated behaviour) with $E_{\rm a}$=32 meV. A good agreement is found in a large temperature range down to about 70-80 K. Inset: Temperature dependence of resistivity in an extended temperature range. (b) $ln\rho$ versus $T^{-1/4}$ for the temperature range below 70 K, showing the fit to a 3D Variable Range Hopping (VRH) model.

Supplementary Note 7: Intermediate state of NNO/STO film

In Supplementary Fig. 9, the resistivity data of a 10 nm film on DSO (same data as in Supplementary Fig. 8) is plotted together with the fit to the NNH model with E_a= 32 meV, which is valid for temperature above about 70-80 K. In addition, the data for another two films of the same thickness under low strain (on LAO) and intermediate strain (on STO) are also shown. The film on STO displays similar behaviour as the film on DSO in the insulating phase: a NNH regime at $T > 80$ K. It is interesting to notice that these films under intermediate strain (on STO) show a magnitude of the resistivity in the metallic state and a T_{MI} that are in between those of the film on DSO and the film on LAO. In addition, the slope of the resistivity of the film on STO seems to approach that of the film on LAO at the largest temperatures.

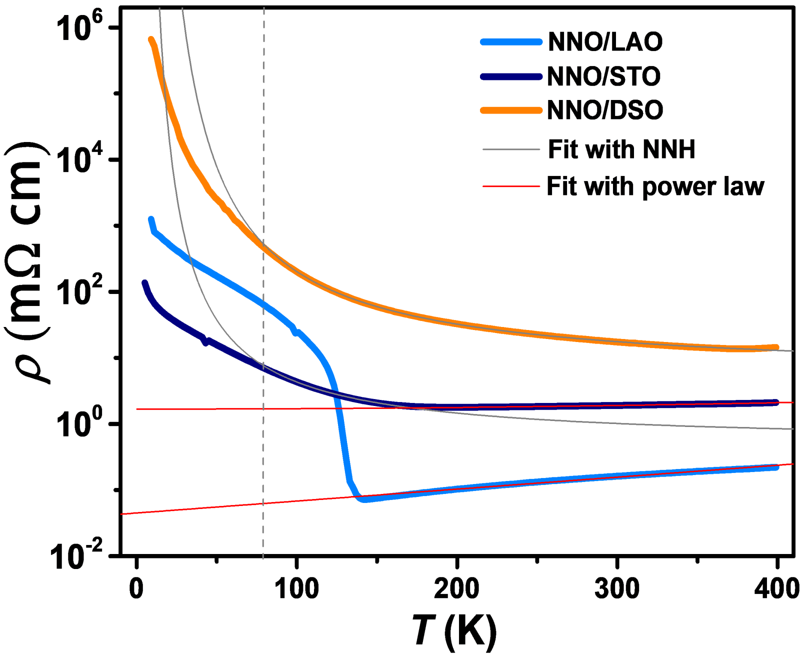

Supplementary Figure 9. Fit to resistivity with various models. ρ(T) curves of 10 nm NNO films grown on LAO, STO, and DSO substrates. The vertical dashed line indicates the temperature of crossover from the Variable Range Hopping (VRH) (fit not shown) to the Near Neighbours Hopping (NNH) regimes. The solid grey lines are the best fits in the NNH regime with activation energies of E_a= 32 meV and E_a= 20 meV, for the samples on DSO and on STO, respectively. The red solid lines are the best fits with power law in the metallic regime of the NNO films grown on LAO and STO substrates.

References

[1] Philips'Gloeilampenfabrieken, O. A method of measuring specific resistivity and hall effect of discs of arbitrary shape. *Philips Res. Rep* **13**, 1–9 (1958).

[2] Preziosi, D., Sander, A., Barthélémy, A. & Bibes, M. Reproducibility and off-stoichiometry issues in nickelate thin films grown by pulsed laser deposition. *AIP Adv.* **7**, 015210 (2017).

[3] Mott, N. Conduction in non-crystalline materials. *Oxford University Press, Walton Street, Oxford OX 2 6 DP, UK.* (1987).

[4] Issai, S. *Is hopping a science?: selected topics of hopping conductivity* (World Scientific, 2015).
